# Blood pressure and Alzheimer's disease: A review of meta-analysis

**DOI:** 10.3389/fneur.2022.1065335

**Published:** 2023-01-11

**Authors:** Olalla Sáiz-Vazquez, Alicia Puente-Martínez, Joaquín Pacheco-Bonrostro, Silvia Ubillos-Landa

**Affiliations:** ^1^Department of Occupational Therapy, Faculty of Health Science, University of Burgos, Burgos, Spain; ^2^Department of Social Psychology and Anthropology, Faculty of Social Sciences, University of Salamanca (USAL), Salamanca, Spain; ^3^Department of Applied Economy, Faculty of Economics and Business Sciences, University of Burgos, Burgos, Spain; ^4^Department of Social Psychology, Faculty of Health Science, University of Burgos, Burgos, Spain

**Keywords:** Alzheimer's disease, blood pressure, systo-diastolic hypertension, risk factor, meta-analysis

## Abstract

**Background:**

Alzheimer's disease (AD) is a neurological disorder of unknown cause, resulting in the death of brain cells. Identifying some of the modifiable risk factors for AD could be crucial for primary prevention and could lead to a reduction in the incidence of AD.

**Objective:**

This study aimed to perform a meta-meta-analysis of studies in order to assess the effect of blood pressure (BP) on the diagnosis of AD.

**Method:**

The search was restricted to meta-analyses assessing high systolic BP (SBP) and diastolic BP (DBP) and AD. We applied the PRISMA guidelines.

**Results:**

A total of 214 studies were identified from major databases. Finally, five meta-analyses (52 studies) were analyzed in this review. Results confirm that high SBP is associated with AD. The exploration of parameters (sex, age, study design, region, and BP measurements) shows that only region significantly moderates the relationship between BP and AD. Asian people are those whose SBP levels >140 mmHg are associated with AD. BP is associated with AD in both people aged ≤65 years and those aged ≥65 years and in cross-sectional and longitudinal studies. In the case of DBP, only women are at a higher risk of AD, particularly when its levels are >90.

**Conclusion:**

SBP is associated with both cerebrovascular disease and AD. Therefore, future studies should use other uncontrolled factors, such as cardiovascular diseases, diabetes, and stroke, to explain the relationship between SBP and AD.

## 1. Introduction

There are 55 million people affected by dementia worldwide (1). Alzheimer's disease (AD) is the most common cause of dementia, accounting for up to 75% of all dementia cases (2). The prevalence of AD increases every year in individuals between the ages of 65 and 85 years (3), and by the year 2050, the worldwide prevalence of AD will grow four-folds, to 106.8 million (range 47.2–221.2) (4). While between the ages of 65 and 74 years, about 10% of people have AD, and in those over 85 years old, the risk increases by 50% (3). According to estimates by the World Health Organization (WHO), the projected global prevalence of AD by 2050 will increase by 110% from 2010 (5).

Alzheimer's disease is a neurological disorder of unknown cause, resulting in the death of brain cells (3). AD is the most common cause of cognitive impairment (6). AD is characterized by hallmark pathological changes such as extracellular Aβ plaques and intracellular neurofibrillary pathology, which selectively affect specific subclasses of neurons and brain circuits. While dementia is a general term, Alzheimer's disease is a specific brain disease. It is marked by symptoms of dementia that gradually get worse over time (7). Dementia is a rather broad syndrome of global cognitive decline. However, AD first affects the part of the brain associated with specific cognitive functions, such as language (aphasia), motor skills (apraxia), and perception (agnosia) (8, 9). Moreover, in AD, early symptoms often include changes in memory, thinking, and reasoning skills (10).

Some of the first symptoms that occur with AD (neuropsychiatric) are a direct cause of early institutionalization (11). In AD, there is an identity loss (12) and worsening in the physical and social areas (11), along with the progressive deterioration of basic cognitive (episodic memory, linguistic, and spatial orienting) and executive functions (inhibitory abilities and the visuospatial functions) (13). Emotional and mental health problems (e.g., delusions and hallucinations, abnormal behaviors, or physical violence and hitting) are common, cause distress to caregivers, and may be amenable to treatment (14, 15). All these symptoms affect the quality of life and activities of daily living in individuals diagnosed with this disease (15).

The most important non-modifiable risk factor for developing AD is age. Many cardiovascular risk factors increase with age, such as high blood pressure (BP), which, in turn, could affect the mechanisms that lead to impairment in the brain (16).

According to Ballard et al. (17), the development of dementia is associated with not only genetic factors but also acquired factors (i.e., hypertension) that could predict a higher risk of AD. In this study, we particularly focused on analyzing high BP as a risk factor for the development of AD (18, 19). The overall prevalence of high BP in adults is 25%, with more than 50% of those individuals over 60 years (20). Vascular risk factors like BP could change the anatomy of the human body by modifying vascular walls or causing ischemia and cerebral hypoxia, which may consequently lead to the development of AD (21). Furthermore, BP could generate dysfunction in the blood–brain barrier, which has been associated with the genesis of AD (22). Studies on the relationship between BP and AD have yielded inconsistent results, showing an association between AD and high BP, or no significant association between these variables (23–25). For example, Mielke found that systolic hypertension was associated with an increased risk of AD. However, the authors did not find an association between diastolic hypertension and AD (22).

Findings also established that the association between AD and hypertension was determined by age of onset (early-onset AD ≤ 65 years and late-onset AD ≥ 65 years). In fact, AD has been classified as presenile or early onset (≤ 65 years) and as senile or late onset (≥65 years) that tend to be sporadic and slow moving (26). However, it is still not clear in the current literature whether age moderates the relationship between BP and AD. Indeed, some researchers have indicated that elevated BP occurring in either middle age or late life may be involved in the development of AD (23, 27, 28). Also, one study concluded that high systolic BP (SBP) and diastolic BP (DBP) were related to worse cognitive function for persons aged 65–74 years. However, in older age (≥75), higher SBP and DBP were related to adequate cognitive function (29).

Other studies have studied the relationship between hypertension and gender. Gillis and Sullivan (30) concluded that women are more likely to be prehypertensive than men. Furthermore, Anstey et al. (31) concluded that hypertension in middle-aged women was associated with greater cognitive impairment and AD. However, recent studies have shown that the prevalence of hypertension is higher in men before the sixth decade of life, although it increases in women after menopause (32).

Related to regions due to the high incidence of hypertension in developed countries, studies are aimed at prevention strategies (33, 34). In addition, the earlier onset and more aggressive development of AD in the young population have been identified as risk factors for hypertension in these countries (35).

The literature refers to various degrees of hypertension. This study was based on the cutoff points established by the International Society of Hypertension (ISH) (36). On the one hand, the ISH establishes the following measures for SBP: elevated (130–139 mmHg), grade 1 (140–159 mmHg), and grade 2 (160–179 mmHg). On the other hand, there are also three cutoff measurements for DBP: elevated (85–89 mmHg), grade 1 (90–99 mmHg), and grade 2 (100–109 mmHg) (36, 37). Mielke et al. (38) concluded that SBP measurements greater than 160 mmHg were associated with greater cognitive impairment in the elderly, which may lead to AD. Similarly, according to Launer et al. (23), elevated midlife SBP > 160 mmHg and DBP ≥ 90 mmHg were particularly associated with an increased risk of AD.

Furthermore, longitudinal (39, 40) and cross-sectional (41, 42) studies have been used to identify risk factors and elucidate some characteristics of AD. To this end, we aggregated data from longitudinal and cross-sectional studies and used meta-analytic equation modeling to test for causal relationships. One major advantage of meta-analytic equations is that it allows an integration of the given data from all studies into one model and specify models that have not been tested in the primary studies (43).

Based on the results and evidence of other articles and meta-analyses, we aimed to perform a meta-meta-analysis of longitudinal and cross-sectional studies to test the association between BP (high SBP and high DBP) and the risk of AD. We also aimed to pool findings separately from cross-sectional and longitudinal studies and assess the effect of BP on the risk of subsequent diagnosis of AD.

## 2. Materials and methods

### 2.1. Data collection

The search was restricted to meta-analyses assessing high SBP and DBP and AD. We applied the Preferred Reporting Items for Systematic Reviews and Meta-Analyses (PRISMA) guidelines (44). The literature searches were carried out in five electronic databases, including ISI Web of Science, Scopus, PubMed, Elsevier Science Direct, and Google Scholar. No publication date was set. The list of keywords was generated through a system of successive approximations: “blood pressure” and “Alzheimer's disease” and “meta-analysis.” A Google Scholar search was also performed but was limited to the title. The literature search was carried out in English and Spanish.

### 2.2. Inclusion criteria

The procedures applied to carry out this meta-meta-analysis were as follows: (1) search and selection of meta-analyses assessing high SBP and DBP and AD and (2) selection of primary studies contained in the meta-analyses and the deletion of duplicates.

Meta-analyses and primary studies that met each of the following criteria were selected: (1) meta-analysis and primary studies that measured the relationship between hypertension (high SBP and DBP) and the risk of AD; (2) meta-analysis and primary studies reported data that allowed the estimation of a pooled effect size; (3) meta-analysis and primary studies that diagnosed AD through clinical examination, using defined diagnostic criteria, DSMV (9) and NINCDS-ADRDA (45); (4) meta-analysis and primary studies that reported the sample size; and (5) meta-analysis and primary studies written in English or Spanish.

To avoid bias in eligible studies, all abstracts were independently reviewed by two investigators (O.S. and A.P.). After excluding all irrelevant abstracts, the remaining articles were analyzed, and data precision was examined in detail. In meta-analysis where relevant data were lacking (*k* = 1), the authors were contacted to request additional data to be subsequently added to the meta-analysis. Then, duplicate reports were excluded to pool the primary studies. After all meta-analyses and primary studies were selected, a third researcher independently extracted the highlighted data (S.U.). Information on all data collected from the primary studies included in the meta-analysis is presented in the Supplementary Table 1.

### 2.3. Quality assessment

The qualities of the meta-analyses were independently coded by two co-authors using the 11-item Assessment of Multiple Systematic Reviews (AMSTAR) tool (46), which has been shown to have a good inter-rater agreement, reliability, and content validity (46, 47). Total scores for the meta-analyses were calculated as the sum of the 11 items on a binary scale. Quality classifications were set as low quality (0–4), moderate quality (5–8), and high quality (9–11).

### 2.4. Statistical analysis

Initially, we reported the associations between hypertension and AD for each primary study included in the previous meta-analysis (see Supplementary material).

Then, for this review of meta-analyses, first, we calculated the cumulative incidence ratio [or log risk ratio (LnRR)] of AD for both SBP and DBP for each primary study. Second, we identified separate effect sizes for SBP and DBP measurements and their relationships with the risk of AD. Third, study outcomes were grouped according to the definition of BP (SBP or DBP) and the measurement of hypertension established by the ISH: (1) SBP: elevated (130–139 mmHg), grade 1 (140–159 mmHg), and grade 2 (160–179 mmHg), and (2) DBP: elevated (85–89 mmHg), grade 1 (90–99 mmHg), and grade 2 (100–109 mmHg) (36, 37). Heterogeneity between study samples was assessed using Cochran's *Q* statistic (48). The *I*^2^ statistic was calculated to express the fraction of variation between studies that was due to heterogeneity. The *I*^2^ statistic explains the percentage of variance in the observed effects due to variance in the true effects. An *I*^2^ value < 25% was considered low heterogeneity, between 25 and 50% was considered moderate heterogeneity, and >50% was considered high heterogeneity (48). Statistical significance was set at *p* ≤ 0.05. Data were analyzed using Comprehensive Meta-Analysis version 3.1 (Biostat Inc, NJ, USA) (49). Additionally, to test for the possibility of publication bias, we computed the Egger regression test. Results revealed no evidence for a publication bias (50).

For each primary study included in the meta-analysis, we calculated the following (see Table 1): (a) *k* or number of studies, (b) effect size, (c) 95% confidence interval (95%CI) of the effect, and (d) *p* (two-tailed significance) (55). We used a random-effect model for the calculation of pooled effect estimates. Then, to assess the heterogeneity of our results, subgroup analyses were performed to examine the differential effects of type of BP: (1) SBP, (2) DBP, and (3) BP (total) on the risk of AD. We did not assume a common among-study variance component across subgroups. High-resolution forest plots were also developed separately with random effects.

**Table 1:** Characteristics of the population of the AD and BP studies.

**References**	**Variable^a^**	**Design^b^**	***K^*c*^***	**Regions (*N*)^d^**	**Sample^e^**	**% F^f^**	**Age^g^**	**SBP/DBP^h^ measure/ mmHg**	**Results**	**Effect size** ^**i**^			**AMSTAR^j^ scores**
										**Effect size (RR)**	**95 % CI** **LLIC**~**ULIC**	***p***	
Lennon et al. (22)	SBP	L (13–22)	6	EU (2), NA (2), AS (2)	AD *n =* 2,208	47.3	M = 56.87	>140 mmHg	> SBP > AD	1.18	1.02–1.35	0.021	10
					HC *n =* 852,683			>160 mmHg	> SBP > AD	1.25	1.06–1.47	0.006	
								>90 mmHg	> DBP > AD^k^				
Xu et al. (51)	SBP	L (1–21)	39	EU (15), NA (20), AS (8), AF (1),	AD *n =* 21,359	50.5	M = 71.8	>140 mmHg	> SBP > AD	0.87	0.70–1.0	0.000	10
					HC *n =* 1,421,593								
	DBP		5		AD *n =* 743			>90 mmHg	> DBP = AD	1.14	0.89–1.39	0.028	
					HC *n =* 11,653								
Meng et al. (52)	SBP	L (10)	1	EU (1)	AD *n =* 79	100	*M* = 45	>140 mmHg	>SBP > AD	1.77	0.93–3.37	0.082	10
					HC *n =* 707								
Guan et al. (53)	SBP	L (2–27)	4	EU (2), NA (1), AS (1)	AD *n =* 176	56.3	40–92	>160 mmHg	>SBP and DBP =AD	1.01	0.87–1.18	0.850	9
	DBP				HC *n =* 7,283			>85 mmHg					
Wang et al. (54)	SBP	T	2	EU (1), NA (1)	AD *n =* 385	39	< 65	>140 mmHg	>SBP = AD	1.50	0.56–4.04	0.036	10
					HC *n =* 3,626			>160 mmHg					
							≥65	>160 mmHg	>SBP = AD	1.00	0.79–1.25	0.180	
							65–75	>160 mmHg	>SBP = AD	1.01	0.66–1.53	0.215	
							75–85	>160 mmHg	>SBP > AD	1.07	0.63–1.82	0.052	
	DBP		2	EU (1), NA (1)	AD *n =* 385		< 65	>90 mmHg	–	1.70	0.80–3.60	–	
					HC *n =* 3626		≥65	>90 mmHg	>DBP = AD	0.75	0.43–1.32	0.066	
							65–75	>85 mmHg	>DBP = AD	0.71	0.30–1.67	0.616	
							75–85	>90 mmHg	>DBP = AD	0.52	0.32–0.85	0.267	

^a^Variable: SBP, systolic blood pressure; DBP, diastolic blood pressure.

^b^Design: T, cross-sectional; L, longitudinal.

^c^K: Number of studies.

^d^Regions: N, number of independent studies; EU, European Union; NA, North America; AS, Asia; AF, Africa.

^e^Sample: AD, participants with Alzheimer's disease; HC, health control participants.

^f^%F: percentage of women.

^g^M, mean of age.

^h^ Study outcomes were grouped according to the measurement of hypertension: (1) SBP > 140 mmHg and >160 mmHg, (2) DBP > 85 mmHg and 90 mmHg [reference guides: (36, 37)].

^i^ CI: 95% confidence interval; RR: risk ratio.

^j^ AMSTAR, Assessing the Methodological Quality of Systematic Reviews. https://amstar.ca/Amstar_Checklist.php.

^k^ Given that two studies used odds ratios and the others hazard ratios, the authors could not compute summary estimates.

Additionally, moderating variables were selected based on substantive considerations and the availability of data across studies included in the meta-analysis. We anticipated interstudy heterogeneity as there was some variation between studies according to the study design (longitudinal *k effect size* = 29 vs. cross-sectional *k effect size* = 46) and the measures of SBP (>140 mmHg *k effect size* = 52 and >160 mmHg *k effect size* = 8) and DBP (>85 mmHg *k effect size* = 2 and >90 mmHg *k effect size* = 9). Finally, we also considered whether age at exposure assessment (early age of onset ≤ 65 *k effect size* = 39 vs. late age of onset or ≥65 *k effect size* = 36) could account for heterogeneity in associations. When possible, we used separate summary measures for early- and late-life measures of BP. Otherwise, BP in early life or late life was defined according to the mean of age. Moreover, we also analyzed the sex (male or female) in the different BP measurements. In the same line, we also analyzed the continent where the sample was recruited (Europe, Asia, and North America) in the different BP measurements.

## 3. Results

A total of 214 studies were identified from major databases: 61 in ISI Web of Science, 55 in Scopus, 17 in PubMed, 79 in Elsevier Science Direct, and 2 in Google Scholar. In total, 189 articles were excluded from this review for various reasons: (a) *k* = 89 were duplicates and (b) *k* = 100, in which no information was provided on the relationship between BP and AD.

**Figure 1:**
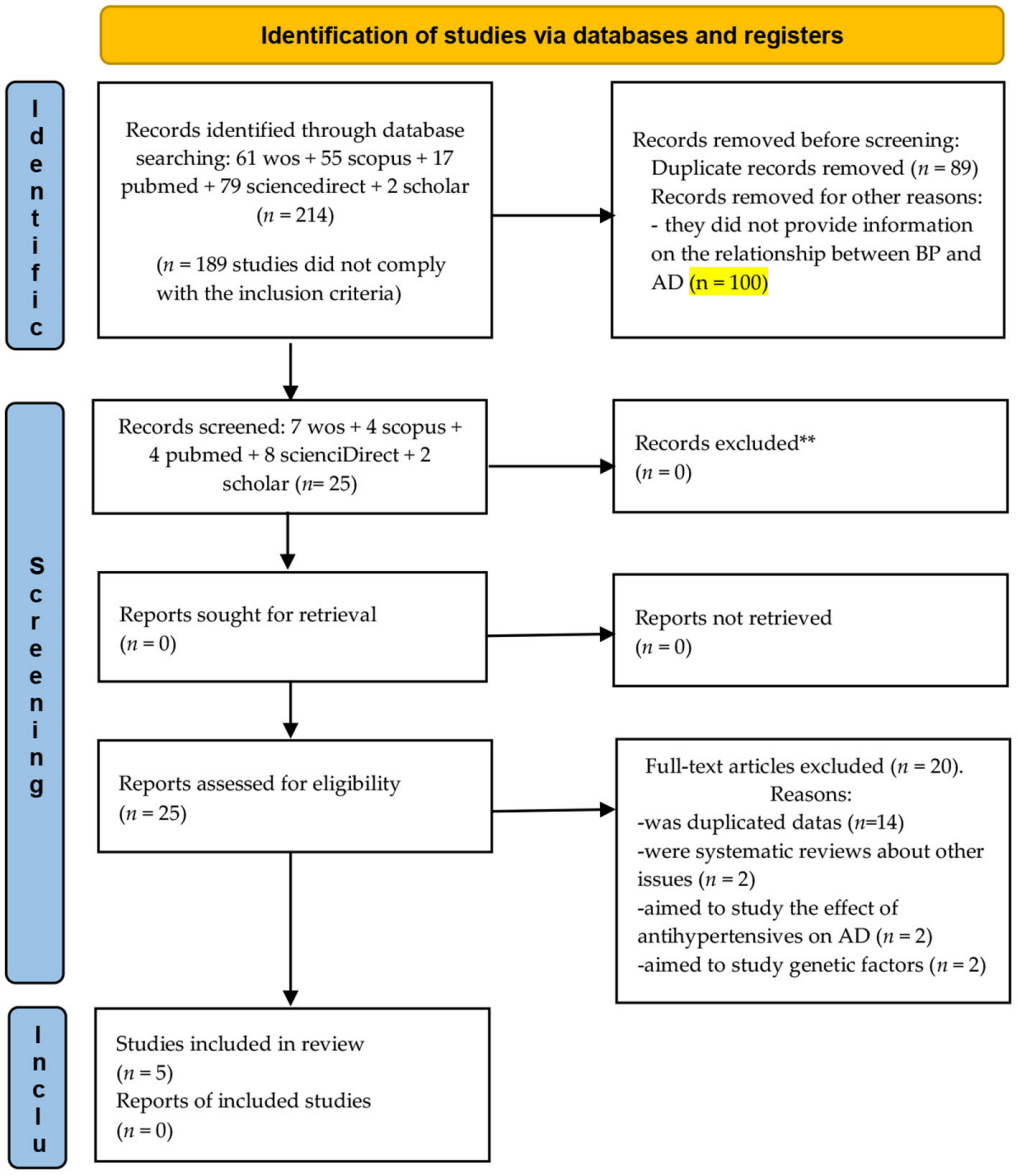
Flowchart depicting the selection of articles for our meta-analysis. From Page et al. (56).

A total of 25 meta-analyses were eligible for inclusion in this review of meta-analyses. Of these meta-analyses, 20 were excluded: (a) *k* = 14 studies were duplicated data; (b) *k* = 2 were systematic reviews about other issues; (c) *k* = 2 aimed to study the effect of antihypertensives on AD; and (d) *k* = 2 aimed to study genetic factors (Figure 1).

Table 1 summarizes key features of the included primary diagnosis, design, number of primary studies, regions of origin of the study, sample size, gender, mean age, results, effect sizes of the relationships between BP and AD, and AMSTAR scores. Although the meta-meta-analyses were based on the criteria established by ISH, the studies only showed values for the following cutoff points: SBP (>140 mmHg and >160 mmHg) and DBP (>85 mmHg and >90 mmHg). Eggers' test was not significant: the intercept (B0) is 0.47, Se = 0.28, 95%CI (−0.09, 1.04), with *t* = 1.65, df = 73, indicating no publication bias.

### 3.1. BP and AD: Heterogeneity analysis

A total of 75 effect sizes were extracted from a total of five meta-analyses that included *k* = 52 primary studies. Also, 60 effect sizes provided information about high SBP and risk of AD (80%); *k* = 11 about high DBP (14.7%); and *k* = 4 about the combined effect (5.3%) (Supplementary Table 1).

For the pooling LnRR analysis, we analyzed primary studies. The total effect size was LnRR = 0.07, Se = 0.02 (0.031, 0.125), Z = 3.27, *p* = 0.001, and heterogeneity was high (Qb = 415.56, df = 74, *p* = 0.0000; *I*^2^ = 82.19). These findings suggest that heterogeneity of effect may be present in some analyses.

### 3.2. Systolic blood pressure and AD

**Figure 2:**
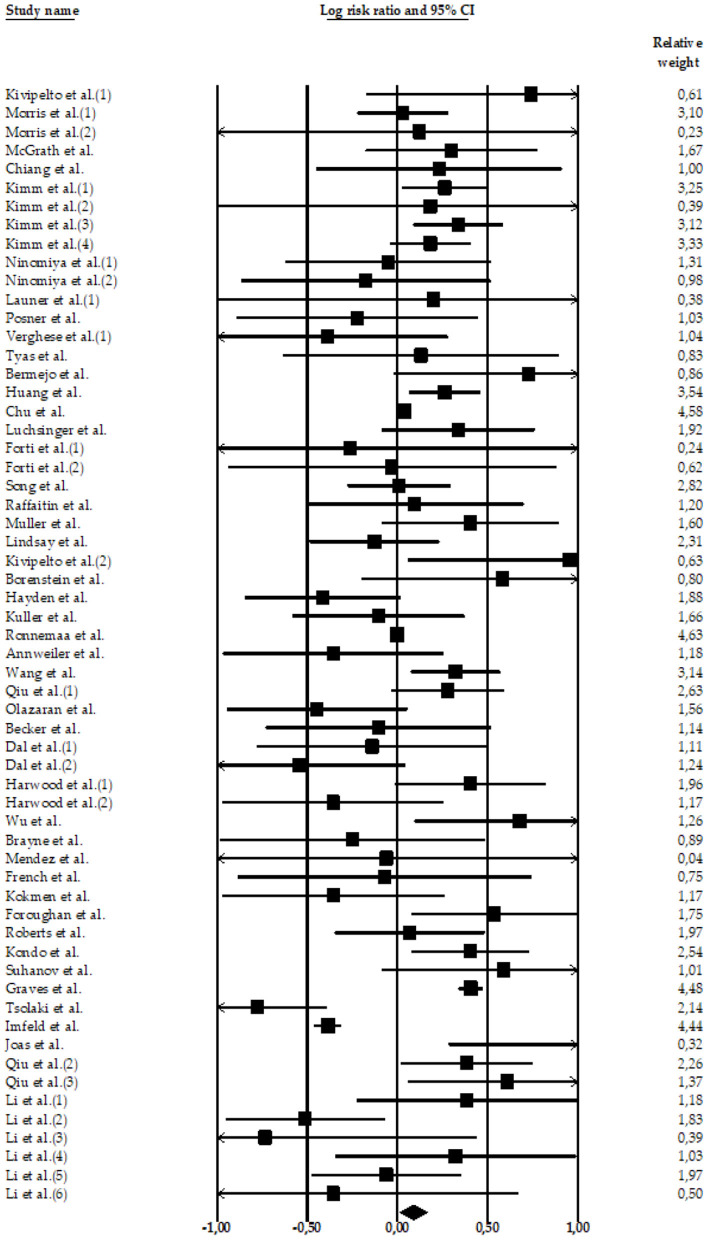
Forest plot of the meta-analysis of incidence rates of AD in participants with high SBP. Individual and pooled estimates of the association between measures of hypertension and AD. The size of the box representing the point estimate for each study in the forest plot is proportional to the contributing weight of that study estimate to the summary estimate.

**Figure 3:**
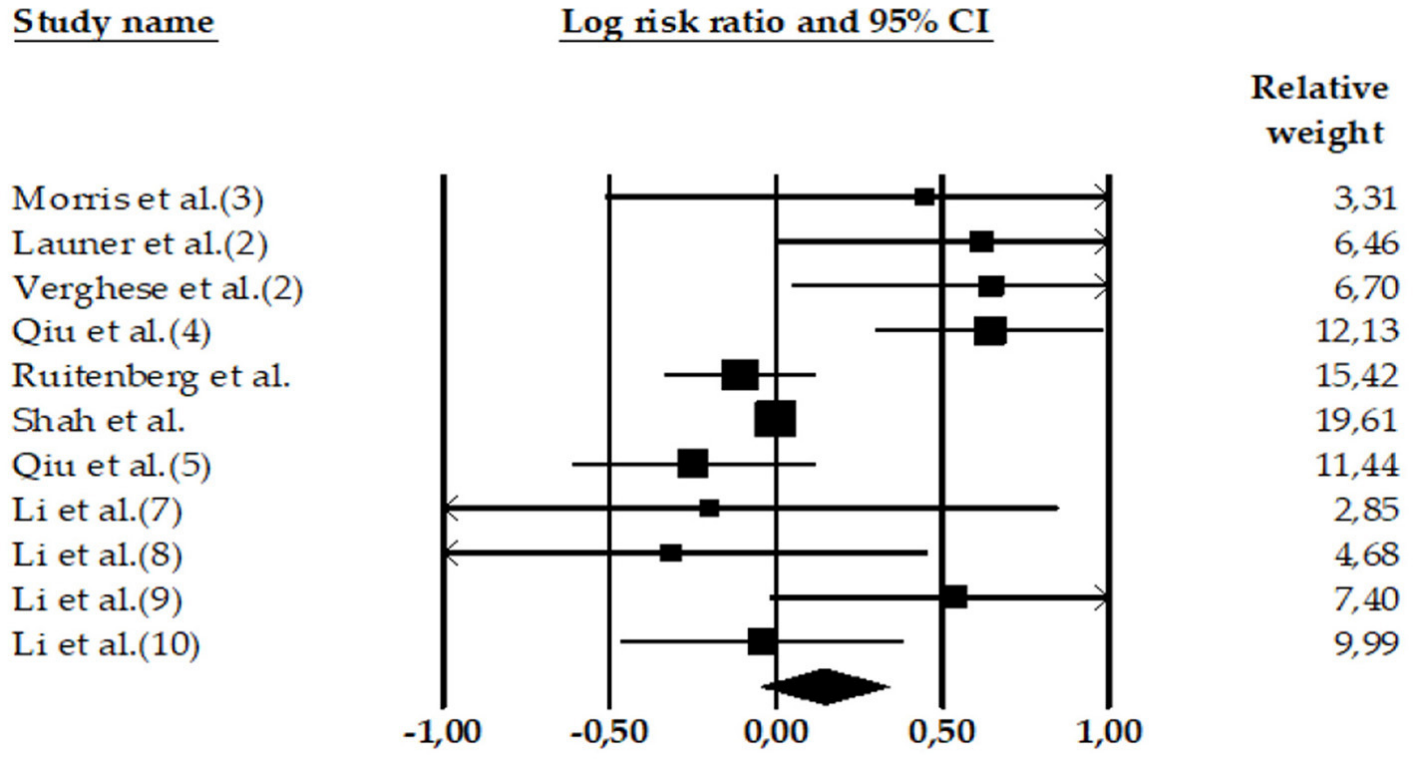
Forest plot of the meta-analysis of incidence rates of AD in participants with high DBP. Individual and pooled effect estimates of the association between DBP hypertension and AD. The size of the box representing the point estimate for each study in the forest plot is proportional to the contributing weight of that study estimate to the summary estimate.

**Figure 4:**
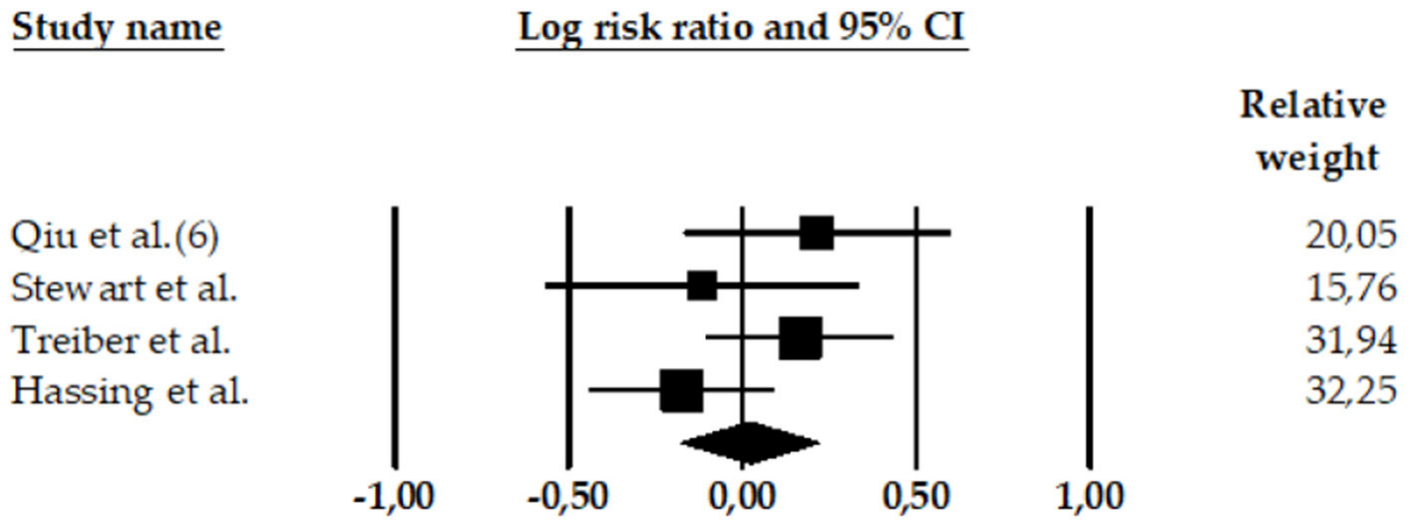
Forest plot of the meta-analysis of rates of AD in participants with high BP (high SBP and high DBP). The size of the box representing the point estimate for each study in the forest plot is proportional to the contributing weight of that study estimate to the summary estimate.

Four meta-analyses examined the relationship between high SBP and AD. The meta-analyses carried out by Lennon et al. (22) (*k* = 11 effect sizes; *N* = 7,666*; n* = 1,520 participants with AD and high SBP*; nHC* = 6,146 HC participants), Xu et al. (51) (*k* = 40 effect sizes; *N* = 1,443,213*; n* = 17,113 participants with AD and high SBP*; n* = 1,426,100 HC participants), Meng et al. (52) (*k* = 1 effect size; *N* = 786; *n* = 79 participants with AD and high SBP; *n* = 707 HC participants), and Wang et al. (54) (*k* = *8* effect sizes; *N* = 5,885; *n* = 385 participants with AD and high SBP; *n* = *5*,500 HC participants) compared HC and AD subjects with high SBP. Only two of them (22, 52) found significant associations between high SBP and the risk of AD (Figures 2–4).

The total random effect of the high SBP value was *k* = 60 effect sizes; *N* = 1,457,550 participants; *nAD* = 19,097 participants; *nHC* = 1,438,453 (LnRR = 0.09, 95%CI = 0.013–0.166, *Z* = 2.28, *p* = 0.022) (see Table 2). The heterogeneity was high: Q-value= 380.08, df = 59, and *I*^2^ = 84.

**Table 2:** Individual and pooled estimates of the association between high SBP and AD.

**References**		**Statistics for each study**						
	**Sample**	***LnRR***	***Se***	***Ve***	***LLIC***	***ULIC***	***Z***	***p***
**Lennon et al**. **(****22****)**								
Kivipelto et al. (1) (18)	AD *n =* 48	0.74	0.47	0.22	−0.174	1.658	1.59	0.113
	HC *n* = 1,400							
Morris et al. (1) (25)	AD *n* = 324	0.03	0.13	0.02	−0.221	0.280	0.23	0.817
	HC *n* = 378							
Morris et al. (2) (25)^a^	AD *n* = 54	0.12	0.79	0.63	−1.430	1.674	0.15	0.877
	HC *n* = 378							
McGrath et al. (57)	AD *n =* 81	0.30	0.24	0.06	−0.174	0.775	1.24	0.215
	HC *n* = 1,440							
Chiang et al. (58)	AD *n =* 64	0.23	0.35	0.12	−0.448	0.910	0.67	0.505
	HC *n* = 292							
Kimm et al. (1) (59)	AD *n =* 282	0.26	0.12	0.01	0.030	0.495	2.21	0.027
	HC *n* = 821							
Kimm et al. (2) (59)	AD *n =* 164	0.18	0.60	0.36	−1.000	1.364	0.30	0.762
	HC *n* = 821							
Kimm et al. (3) (59)^a^	AD *n =* 274	0.34	0.13	0.02	0.088	0.584	2.66	0.008
	HC *n* = 821							
Kimm et al. (4) (59)^a^	AD *n =* 206	0.18	0.11	0.01	−0.041	0.405	1.60	0.109
	HC *n* = 821							
Ninomiya et al. (1) (60)	AD *n =* 6	−0.05	0.29	0.08	−0.619	0.516	−0.18	0.859
	HC *n* = 149							
Ninomiya et al. (2) (60)^a^	AD *n =* 17	−0.17	0.35	0.12	−0.865	0.516	−0.50	0.621
	HC *n* = 177							
**Total (22)**		**0.20**	**0.06**	**0.00**	**0.090**	**0.307**	**3.58**	**0.000**
**Xu et al**. **(****51****)**								
Launer et al. (1) (27)	AD *n =* 81	0.20	0.61	0.37	−0.996	1.394	0.33	0.744
	HC *n* = 2.137							
Posner et al. (24)	AD *n =* 257	−0.22	0.34	0.12	−0.892	0.446	−0.65	0.513
	HC *n* = 1.259							
Verghese et al. (1) (61)	AD *n =* 65	−0.39	0.34	0.11	−1.049	0.278	−1.14	0.255
	HC *n* = 406							
Tyas et al. (39)	AD *n =* 35	0.13	0.39	0.15	−0.634	0.897	0.34	0.737
	HC *n* = 685							
Bermejo et al. (62)	AD *n =* 113	0.73	0.38	0.15	−0.020	1.475	1.91	0.056
	HC *n* = 3.824							
Huang et al. (63)	AD *n =* 612	0.26	0.10	0.01	0.064	0.460	2.60	0.009
	HC *n* = 142.744							
Chu et al. (64)	AD *n =* 10	0.04	0.02	0.00	0.009	0.069	2.54	0.011
	HC *n* = 153							
Luchsinger et al. (65)	AD *n =* 246	0.34	0.22	0.05	−0.087	0.760	1.56	0.120
	HC *n* = 1.138							
Forti et al. (1) (66)	AD *n =* 18	−0.26	0.77	0.60	−1.777	1.254	−0.34	0.735
	HC *n* = 466							
Forti et al. (2) (66)	AD *n =* 30	−0.03	0.46	0.21	−0.939	0.878	−0.07	0.948
	HC *n* = 238							
Song et al. (67)	AD *n =* 416	0.01	0.15	0.02	−0.276	0.296	0.07	0.946
	HC *n* = 2.790							
Raffaitin et al. (68)	AD *n =* 134	0.10	0.31	0.10	−0.509	0.700	0.31	0.757
	HC *n* = 7.087							
Muller et al. (69)	AD *n =* 147	0.41	0.25	0.06	−0.085	0.896	1.62	0.105
	HC *n* = 1833							
Lindsay et al. (70)	AD *n =* 194	−0.13	0.18	0.03	−0.486	0.231	−0.70	0.485
	HC *n* = 4.088							
Kivipelto et al. (1) (71)	AD *n =* 48	0.96	0.46	0.21	0.060	1.851	2.09	0.037
	HC *n* = 1.449							
Borenstein et al. (72)	AD *n =* 90	0.58	0.40	0.16	−0.196	1.361	1.47	0.143
	HC *n* = 1.859							
Hayden et al. (73)	AD *n =* 104	−0.42	0.22	0.05	−0.847	0.016	−1.89	0.059
	HC *n* = 3.264							
Kuller et al. (74)	AD *n =* 330	−0.11	0.24	0.06	−0.582	0.372	−0.43	0.665
	HC *n* = 2.807							
Ronnemaa et al. (75)	AD *n =* 127	0.00	0.09	0.01	−0.182	0.182	0.00	1.000
	HC *n* = 2.268							
Annweiler et al. (76)	AD *n =* 70	−0.36	0.31	0.10	−0.968	0.254	−1.14	0.253
	HC *n* = 498							
Wang et al. (77)	AD *n =* 8.488	0.32	0.13	0.02	0.076	0.568	2.57	0.010
	HC *n* = 1.230.400							
Qiu et al. (1) (78)	AD *n =* 333	0.28	0.16	0.03	−0.034	0.590	1.74	0.081
	HC *n* = 1.301							
Olazaran et al. (79)	AD *n =* 68	−0.45	0.26	0.07	−0.946	0.054	−1.75	0.080
	HC *n* = 1.376							
Becker et al. (80)	AD *n =* 48	−0.11	0.32	0.10	−0.729	0.518	−0.33	0.740
	HC *n* = 288							
Dal et al. (1) (81)	AD *n =* 40	−0.14	0.32	0.11	−0.775	0.496	−0.43	0.668
	HC *n* = 576							
Dal et al. (2) (81)	AD *n =* 67	−0.54	0.30	0.09	−1.134	0.045	−1.81	0.070
	HC *n* = 781							
Harwood et al. (1) (82)	AD *n =* 202	0.41	0.21	0.05	−0.011	0.822	1.91	0.056
	HC *n* = 392							
Harwood et al. (2) (82)	AD *n =* 188	−0.36	0.31	0.10	−0.969	0.256	−1.14	0.254
	HC *n* = 84							
Wu et al. (83)	AD *n =* 201	0.68	0.30	0.09	0.095	1.261	2.28	0.023
	HC *n* = 391							
Brayne et al. (84)	AD *n =* 18	−0.25	0.37	0.14	−0.983	0.486	−0.66	0.507
	HC *n* = 340							
Mendez et al. (85)	AD *n =* 50	−0.06	2.02	4.07	−4.015	3.891	−0.03	0.976
	HC *n* = 407							
French et al. (86)	AD *n =* 76	−0.07	0.42	0.17	−0.887	0.742	−0.17	0.861
	HC *n* = 102							
Kokmen et al. (87)	AD *n =* 203	−0.36	0.31	0.10	−0.972	0.258	−1.14	0.256
	HC *n* = 415							
Foroughan et al. (88)	AD *n =* 42	0.54	0.23	0.05	0.078	0.995	2.30	0.022
	HC *n* = 115							
Roberts et al. (89)	AD *n =* 151	0.07	0.21	0.04	−0.348	0.483	0.32	0.750
	HC *n* = 264							
Kondo et al. (90)	AD *n =* 60	0.41	0.16	0.03	0.082	0.729	2.46	0.014
	HC *n* = 120							
Suhanov et al. (91)	AD *n =* 127	0.59	0.34	0.12	−0.086	1.262	1.71	0.087
	HC *n* = 260							
Graves et al. (92)	AD *n =* 18	0.43	0.03	0.01	0.339	0.472	11.90	0.000
	HC *n* = 340							
Tsolaki et al. (93)	AD *n =* 65	−0.77	0.19	3.86	−1.161	−0.391	−3.94	7.829
	HC *n* = 69							
Imfeld et al. (94)	AD *n =* 3.541	−0.38	3.75	1.41	−0.459	−0.312	−10.26	0.000
	HC *n* = 7.086							
**Total (52)**		**0.05**	**0.05**	**0.00**	**−0.038**	**0.146**	**1.16**	**0.246**
**Meng et al**. **(****52****)**								
Joas et al. (95)	AD *n =* 79	1.59	0.67	0.45	0.285	2.902	2.39	0.017
	HC *n* = 707							
**Wang et al**. **(****54****)**								
Qiu et al. (2) (96)	AD *n =* 150	0.61	0.28	0.08	0.060	1.159	2.18	0.030
	HC *n* = 1.270							
Qiu et al. (3) (96)^a^	AD *n =* 124	0.39	0.19	0.03	0.019	0.751	2.06	0.039
	HC *n* = 441							
Li et al. (1) (97)	AD *n =* 14	0.39	0.31	0.10	−0.225	0.995	1.24	0.216
	HC *n* = 530							
Li et al. (2) (97)	AD *n =* 19	−0.51	0.23	0.05	−0.953	−0.069	−2.26	0.024
	HC *n* = 733							
Li et al. (3) (97)	AD *n =* 37	−0.73	0.60	0.36	−1.908	0.440	−1.23	0.220
	HC *n* = 530							
Li et al. (4) (97)^a^	AD *n =* 31	0.32	0.34	0.12	−0.346	0.990	0.95	0.345
	HC *n* = 733							
Li et al. (5) (97)^a^	AD *n =* 4	−0.06	0.21	0.04	−0.476	0.352	−0.29	0.770
	HC *n* = 733							
Li et al. (6) (97)^a^	AD *n =* 6	−0.36	0.52	0.27	−1.384	0.670	−0.68	0.496
	HC *n* = 530							
**Total** **(****55****)**		**0.08**	**0.16**	**0.03**	**−0.241**	**0.399**	**0.48**	**0.629**
**Total random**		**0.09**	**0.04**	**0.00**	**0.013**	**0.166**	**2.28**	**0.022**

^a^Measures SBP > 160.

### 3.3. Diastolic blood pressure and AD

Three meta-analyses showed the relationship between DBP and AD: Lennon et al. (22) (*k* = 1 effect size; *N* = 378; *n* = 78 with AD and high DBP; *n* = 300 HC participants), Xu et al. (51) (*k* = 5 effect sizes; *N* = 12,225; *n* = 497 with AD and high DBP; *n* = 11,728 HC participants), and Wang et al. (54) (*k* = 5 effect sizes; *N* = 7,745; *n* = 306 with AD and high DBP; *n* = 7,439 HC participants). None of the three meta-analyses show significant associations between high DBP and AD.

Consistently, our results (*k* = 11 effect sizes; *N* = 20,348; *nAD* = 881; HC = 19,467) did not find an association between high DBP and the risk of AD (LnRR = 0.15, 95% CI = −0.045 to 0.338, *Z* = 1.50, *p* = 0.133) (see Table 3). The heterogeneity was high: Q-value = 29.99, df = 10, and *I*^2^ = 66.65.

**Table 3:** Individual and pooled estimates of the association between high DBP and AD.

**References**	***Sample***	**Statistics for each study**						
		***LnRR***	***Se***	***Ve***	***LLIC***	***ULIC***	***Z***	***p***
**Lennon et al**. **(****22****)**								
Morris et al. (3) (25)	AD *n =* 78	0.44	0.49	0.24	−0.513	1.402	0.91	0.363
	HC *n* = 300							
**Xu et al**. **(****51****)**								
Launer et al. (2) (27)	AD *n =* 87	0.62	0.31	0.10	0.005	1.236	1.98	0.048
	HC *n* = 2.137							
Verghese et al. (2) (61)	AD *n =* 65	0.65	0.31	0.09	0.048	1.246	2.12	0.034
	HC *n* = 406							
Qiu et al. (4) (78)	AD *n =* 87	0.64	0.17	0.03	0.303	0.981	3.71	0.000
	HC *n* = 1.301							
Ruitenberg et al. (98)	AD *n =* 107	−0.11	0.11	0.01	−0.331	0.120	−0.92	0.359
	HC *n* = 6.985							
Shah et al. (99)	AD *n =* 151	0.00	0.01	0.00	−0.010	0.010	0.00	1.000
	HC *n* = 899							
**Total** (52)		**0.27**	**0.15**	**0.02**	**−0.019**	**0.554**	**1.83**	**0.068**
**Wang et al**. **(****54****)**								
Qiu et al. (5) (96)	AD *n =* 245	−0.25	0.19	0.03	−0.613	0.116	−1.34	0.182
	HC *n* = 2,249							
Li et al. (7) (97)	AD *n =* 22	−0.20	0.53	0.28	−1.245	0.848	−0.37	0.710
	HC *n* = 2.605							
Li et al. (8) (97)	AD *n =* 28	−0.31	0.39	0.15	−1.086	0.457	−0.80	0.424
	HC *n* = 1.321							
Li et al. (9) (97)^a^	AD *n =* 4	0.54	0.28	0.08	−0.018	1.091	1.90	0.058
	HC *n* = 905							
Li et al. (10) (97)^a^	AD *n =* 7	−0.04	0.22	0.05	−0.464	0.383	−0.19	0.850
	HC *n* = 359							
**Total** **(****54****)**		−0.04	0.15	0.02	−0.339	0.263	−0.25	0.805
**Total random**		**0.15**	**0.10**	**0.01**	**−0.045**	**0.338**	**1.50**	**0.133**

^a^Measures DBP > 90.

### 3.4. High SBP and high DBP studies: Combined effect sizes

A meta-analysis reported a combined effect size for high SBP and high DBP (97). This study (*k* = 4 effect sizes; *N* = 7,494; *n* = 211 with AD and high SBP/DBP; *n* = 7,283 HC participants) found a non-significant association between high SBP and high DBP and AD (LnRR = 0.02, 95% CI = −0.179 to 0.222, Z = 0.21, *p* = 0.835) (see Table 4). The heterogeneity was medium: Q-value = 4.52, df = 3, and *I*^2^ = 33.69.

**Table 4:** Individual and pooled estimates of the association between high BP and AD.

**References**	***Sample***	**Statistics for each study**						
		***LnRR***	***Se***	***Ve***	***LLIC***	***ULIC***	***Z***	***p***
**Guan et al**. **(****53****)**								
Qiu et al. (6) (19)	AD *n =* 75	0.22	0.20	0.04	−0.168	0.599	1.10	0.272
	HC *n* = 719							
Stewart et al. (100)	AD *n =* 35	−0.12	0.23	0.05	−0.566	0.333	−0.51	0.611
	HC *n* = 1.778							
Treiber et al. (101)	AD *n =* 65	0.17	0.14	0.02	−0.103	0.434	1.21	0.227
	HC *n* = 3.634							
Hassing et al. (102)	AD *n =* 36	−0.17	0.14	0.02	−0.441	0.092	−1.28	0.199
	HC *n* = 1.152							
**Total random**		**0.02**	**0.10**	**0.01**	**−0.179**	**0.222**	**0.21**	**0.835**

### 3.5. Subgroup analyses

Results of the subgroup analysis on the primary outcomes are presented in Table 5. Study outcomes were grouped by definition of hypertension and measures of BP (e.g., SBP, DBP, or total BP). Notably, 60 effect sizes examined SBP at both grades (22): 52 effect sizes examined only grade 1 (>140 mmHg) (51, 54) and 8 effect sizes examined only grade 2 (>160 mmHg) (53). Eleven effect sizes examined DBP at both grades: 2 effect sizes examined DBP using a cutoff point of >85 mmHg (51, 54) and 9 effect sizes >90 mmHg. Four effect sizes combined both types of hypertension (53). Moderator analyses were performed comparing effect sizes according to sex (men and women), age (≤ 65 and ≥66), study design (cross-sectional or C and longitudinal or L), and regions (Europe, Asia, and North America).

**Table 5:** Effects of sex, age, design, and regions in different types of SBP (>140 and >160 mmHg) and DBP (>85 and >90 mmHg).

**Group by**		**Statistics for each study**									
		**Effect sizes**	***LnRR***	***Se***	***Ve***	***LLIC***	***ULIC***	***Z***	***p***	***I** ^2^*	***Qb***
**BP (all types)**											
	**Sex**										
	Men	54	0.06	0.04	0.00	−0.023	0.140	1.407	0.159	72.01	1.867, *p* = 0.172
	Women	21	0.16	0.06	0.00	0.041	0.274	2.657	0.008	88.38	
	**Age**										
	≤ 65	36	0.09	0.03	0.00	0.024	0.160	2.645	0.008	58.70	0.280, *p* = 0.596
	≥65	39	0.07	0.03	0.00	0.001	0.132	1.984	0.047	88.11	
	**Design**										
	C	46	0.06	0.03	0.00	0.010	0.120	2.303	0.021	87.61	0.744, *p* = 0.389
	L	29	0.11	0.04	0.00	0.023	0.197	2.484	0.013	36.48	
	**Regions**										
	Europe	23	−0.05	0.03	0.00	−0.113	0.025	−1.244	0.214	87.66	20.65, *p* = 0.0001
	Asia	15	0.19	0.04	0.00	0.115	0.284	4.627	0.000	58.27	
	North America	37	0.11	0.04	0.00	0.038	0.190	2.939	0.003	62.02	
**SBP**											
>140		52	0.08	0.04	0.01	−0.007	0.158	1.786	0.074	86.01	0.948, *p* = 0.330
>160		8	0.19	0.11	0.01	−0.027	0.407	1.720	0.085	3.14	
	**Sex**										
	Men	42	0.08	0.05	0.01	−0.015	0.174	1.649	0.099	67.99	0.107, *p* = 0.744
	Women	18	0.11	0.06	0.01	−0.012	0.221	1.158	0.079	88.94	
>140	Men	35	0.06	0.05	0.01	−0.045	0.162	1.11	0.267	71.87	0.237, *p* = 0.626
	Women	17	0.09	0.06	0.00	−0.025	0.222	1.565	0.118	89.81	
>160	Men	7	0.21	0.11	0.01	−0.009	0.426	1.880	0.060	15.65	0.018, *p* = 0.895
	Women	1	0.18	0.11	0.01	−0.041	0.405	1.601	0.109	0.000	
	**Age**										
	* ≤ 65*	29	0.101	0.07	0.01	−0.034	0.250	1.495	0.135	54.50	0.133, *p* = 0.715
	*≥65*	31	0.07	0.07	0.01	−0.063	0.207	1.040	0.298	90.29	
>140	≤ 65	25	0.08	0.08	0.01	−0.084	0.234	0.927	0.354	49.01	0.000, *p* = 0.987
	≥65	27	0.08	0.07	0.01	−0.067	0.221	1.048	0.295	91.54	
>160	≤ 65	4	0.26	0.10	0.01	0.070	0.455	2.667	0.008	23.26	1.854, *p* = 0.173
	≥65	4	0.01	0.17	0.03	−0.318	0.334	0.047	0.962	0.00	
	**Design**										
	*C*	41	0.06	0.05	0.01	−0.031	0.152	1.294	0.196	88.23	1.336, *p =* 0.248
	*L*	19	0.16	0.07	0.01	0.018	0.302	2.206	0.027	35.78	
>140	C	41	0.06	0.05	0.00	−0.032	0.152	1.290	0.198	88.23	0.517, *p* = 0.472
	L	11	0.14	0.10	0.01	−0.052	0.327	1.425	0.154	50.73	
>160	C	–	–	–	–	–	–	–	–		–
	L	8	0.21	0.07	0.01	0.065	0.356	2.834	0.005	3.14	
	**Regions**										
	Europe	18	0.03	0.09	0.01	−0.148	0.198	0.284	0.777	89.30	5.785, *p =* 0.055
	Asia	14	0.27	0.09	0.01	0.095	0.436	3.044	0.002	60.41	
	North America	28	0.01	0.07	0.01	−0.130	0.152	0.156	0.876	64.11	
>140	Europe	17	0.00	0.09	0.01	−0.187	0.176	0.057	0.955	89.62	5.985, *p* = 0.050
	Asia	11	0.29	0.10	0.01	0.091	0.493	2.854	0.004	63.14	
	North America	24	0.01	0.08	0.01	−0.143	0.160	0.109	0.913	67.66	
>160	Europe	1	0.61	0.28	0.08	0.060	1.159	2.176	0.030	0.00	3.562, *p* = 0.169
	Asia	3	0.23	0.08	0.01	0.067	0.389	2.771	0.006	9.15	
	North America	4	0.01	0.17	0.03	−0.318	0.334	0.047	0.962	0.00	
**DBP**											
>85		2	0.21	0.24	0.06	−0.266	0.680	0.859	0.390	61.98	0.067, *p* = 0.795
>90		9	0.14	0.11	0.01	−0.081	0.358	1.236	0.217	69.65	
	**Sex**										
	Men	8	−0.01	0.06	0.01	−0.13	0.118	−0.109	0.913	39.20	13.37, *p* = 0.0001
	Women	3	0.62	0.15	0.03	0.307	0.927	3.897	0.0001	0.00	
>85	Men	2	0.22	0.29	0.08	−0.344	0.782	0.763	0.446	61.98	–
	Women	–	–	–	–	–	–	–	–	–	
>90	Men	6	−0.02	0.05	0.01	−0.126	0.079	−0.452	0.641	35.53	16.052, *p* = 0.0001
	Women	3	0.62	0.15	0.02	0.321	0.915	4.081	0.0001	0.00	
	**Age**										
	≤ 65	4	0.21	0.18	0.03	−0.133	0.552	1.198	0.231	85.01	0.131, *p* = 0.717
	≥65	7	0.12	0.16	0.03	−0.196	0.442	0.756	0.449	39.41	
>85	≤ 65	–	–	–	–	–	–	–	–	–	–
	≥65	2	0.22	0.29	0.08	−0.344	0.782	0.763	0.446	61.98	
>90	≤ 65	4	0.21	0.18	0.03	−0.147	0.574	1.160	0.246	85.01	0.245, *p* = 0.621
	≥65	5	0.08	0.21	0.04	−0.334	0.485	0.363	0.716	36.35	
	**Design**										
	C	5	0.26	0.14	0.02	−0.015	0.537	1.854	0.064	82.58	1.345, *p* = 0.246
	L	6	0.01	0.17	0.023	−0.317	0.334	0.052	0.958	28.15	
>85	C	–	–	–	–	–	–	–	–		–
	L	2	0.22	0.29	0.08	−0.344	0.782	0.763	0.446	61.98	
>90	C	5	0.26	0.14	0.02	−0.013	0.530	1.864	0.062	82.58	2.450, *p* = 0.118
	L	4	−0.15	0.21	0.05	−0.575	0.282	−0.671	0.502	0.00	
	**Regions**										
	Europe	3	0.12	0.19	0.04	−0.253	0.498	0.638	0.523	87.13	0.074, *p* = 0.786
	Asia	–	–	–	–	–	–	–	–	–	
	North America	8	0.19	0.15	0.02	−0.109	0.487	1.241	0.215	49.06	
>85	Europe	–	–	–	–	–	–	–	–	–	–
	Asia	–	–	–	–	–	–	–	–	–	
	North America	2	0.22	0.29	0.08	−0.344	0.782	0.763	0.446	61.98	
>90	Europa	3	0.12	0.21	0.04	−0.278	0.525	0.604	0.546	87.13	0.041, *p* = 0.840
	Asia	–	–	–	–	–	–	–	–	–	
	North America	6	0.18	0.19	0.04	−0.193	0.554	0.946	0.344	53.09	
**BP (combined effects)**											
	**Sex**										
	Men	4	0.02	0.10	0.01	−0.179	0.222	0.209	0.835	33.68	–
	Women	–	–	–	–	–	–	–	–	–	
	**Age**										
	≤ 65	3	−0.05	0.12	0.02	−0.289	0.192	−0.387	0.669	27.19	0.978, *p* = 0. 323
	≥65	1	0.17	0.18	0.03	−0.182	0.513	0.934	0.350	0.00	
	**Design**										
	C										–
	L	2	0.02	0.10	0.01	−0.179	0.222	0.209	0.835	33.69	
	**Regions**										
	Europe	2	−0.01	0.19	0.04	−0.383	0.383	−0.026	0.979	62.61	0.522, *p* = 0. 770
	Asia	1	−0.12	0.32	0.10	−0.736	0.503	−0.368	0.713	0.00	
	North America	1	0.16	0.26	0.07	−0.339	0.670	0.643	0.520	0.00	

The results of pooling studies that reported RRs for a total score of BP showed that sex, age, and design did not moderate the relationship between hypertension and AD risk (Qb: *p* ≤ 0.50). These results indicate that the risk of AD in participants with hypertension did not change significantly according to sex, age, and study design groups. However, it can be observed that there are significant relationships between different categories of the variables such as sex, age, study design, and AD (*Z*: *p* ≤ 0.50). Findings revealed a significant relationship only between being women and a greater risk of AD (*p* = 0.008). Age was also associated with increased risk of AD in early (*p* = 0.008) and late (*p* = 0.047) age of onset, and this association was also significant in cross-sectional (*p* = 0.021) and longitudinal (*p* = 0.013) studies. Regions moderated the association between BP and AD. The risk of AD was greater in studies that used samples from Asia and North America than those performed in Europe.

Results did not find significant differences in the risk of AD according to the measures of SBP (>140 and >160 mmHg) and DBP (>85 and >90 mmHg). Similarly, sex, age, design, and region did not moderate the relationship between SBP and DBP and the risk of AD, except sex in the case of DBP. Results found that women showed a stronger risk of developing AD than men. It is also observed that only in longitudinal studies and Asia regions, significant associations were found between SBP and AD.

According to measures of SBP (>140 and >160 mmHg), results indicated that SBP had no significant differences in effect sizes on the risk of AD at different sexes, ages, and designs. However, for SBP > 140 mmHg, there was evidence of heterogeneity between regions in RRs of AD. Asian countries showed stronger effect sizes between SBP and risk of AD than European and North American countries. Also, results found that elevated SBP (>160 mmHg) was significantly associated with AD risk in the young elderly (≤ 65), longitudinal studies, and in Europa and Asia.

For DBP (>85 and >90 mmHg), there was evidence of heterogeneity between the sexes. Women with elevated DBP (>90 mmHg) showed a greater risk of AD than men. Furthermore, there were no significant differences in AD risk according to age, design, and region.

Finally, age and region did not moderate the relationship between the combined effects of BP and the risk of AD.

## 4. Discussion

This study analyzes the association between high BP and the risk of AD. This is the first study to evaluate this relationship by identifying previous meta-analyses and analyzing primary studies worldwide. The present study summarized the information on meta-analyses of hypertension (DBP and SBP) and AD and expanded the findings from individual studies. In this study, 52 primary studies and 75 effect sizes were extracted. Furthermore, we included some moderator variables between high DBP and high SBP and AD, such as sex, age, study design, regions, and measures of SBP and DBP.

Overall, results suggest that hypertension is associated with an increased risk of AD (*RR* = 1.08, 95%CI: 1.032, 1.13, *Z* = 3.273, *p* = 0.001). It indicates that the risk of AD increases by 8% for patients with SBP.

In this study, 46 primary studies and 60 effect sizes extracted from four meta-analyses (22, 51–53) confirm the relationship between high SBP and AD (*RR* = 1.09, 95%CI: 1.013, 1.181, *Z* = 2.285, *p* = 0.022). These results indicate that participants with high SBP increase the rate risk of AD by 9% and support findings of previous studies, suggesting that there were consistent demonstrations of a relationship between SBP and the risk of developing AD. In this vein, research demonstrated that high SBP could increase the risk of AD since it could cause neurobiological alterations (deposits of beta-amyloid protein), which lead to lesions in the brain, such as cerebral atrophy, senile plaques, and neurofibrillary tangles, which could be explanatory factors of the development of AD (103, 104). Other studies also suggest that high SBP could cause brain vascular injury, leading to increased flow of blood, cerebral patency, and cerebral amyloid angiopathy which were also associated with a higher risk of AD (105–107). However, our analysis cannot underlie the pathophysiology of AD and could only define SBP as a risk factor.

The relationship between high DBP and AD was studied through *k* = 8 primary studies and eleven effect sizes (three meta-analyses) (22, 51, 54). Findings did not find a significant association between high DBP and the risk of AD. Nevertheless, according to previous studies, these results could be explained by confounding due to associations between BP and advanced disease or other unknown modifiable risk factors (108–110). For instance, secondary diseases, such as obesity, cardiovascular diseases, silent infarcts, and vascular risk factors (111) or type 2 diabetes (103, 108, 109), could be closely related to the development of AD. Hence, in these cases, it is not clear if hypertension is directly related to the risk of AD or whether AD is indirectly motivated by a secondary disease (110). Finally, there was a small number of studies analyzing DBP and AD in comparison with SBP, and in consequence, it is possible that we did not have sufficient statistical power to obtain a significant pooled estimate of the association between DBP and AD.

Related to the combined BP hypertension, only a meta-analysis (53) with four independent studies and effect sizes compared the incidence of AD between subjects with and without hypertension. These studies found that high BP is not associated with an increased risk of AD. This result is contradictory to the general view on the association between risk for AD and hypertension. For example, Guan et al. (53) highlighted that AD and hypertension are independent diseases with some common etiopathogenesis, which is a risk factor in AD.

To explore the influence of other research parameters in the relationship between high SBP and high DBP with AD, we analyzed different moderators: sex, age, study design, and region. This study does not find differences in the risk of AD according to the type of measure of SBP (>140 and >160 mmHg) and DBP (>85 and >90 mmHg). Total scores reveal significant differences between men (*RR* = 0.99, 95%CI: 0.887, 1.125, *Z* = −0.109, *p* = 0.913) and women (*RR* = 1.85, 95% CI: 1.359, 2.527, *Z* = 3.897, *p* = 0.001) (rate risk of AD increases by 85%) in the relationship of high DBP and AD, but not between SBP and AD. Specifically, the data suggest that women with high DBP (>90 mmHg) had an increased risk of AD compared with men (*RR* = 1.86, 95%CI: 1.379, 2.498, *Z* = 16.05, *p* = 0.001), which increase the rate risk of AD by 86%. These results have been shown in previous studies that worked with different samples (women and men), where AD was also associated with high DBP mainly in women (107, 108). For instance, Benetos et al. (112) found that DBP in women is associated with a higher cardiac output, pulse pressure, and heart rate (HR) factors that are related to a higher risk of AD (63.8%).

Total scores of BP show that age is associated with increased risk of AD in the early and late age of onset (*RR* = 1.10, 95%CI: 1.024, 1.174, *Z* = 2.645, *p* = 0.008; *RR* = 1.07, 95%CI: 1.001, 1.141, *Z* = 0.047, *p* = 0.047), with the rate risk of AD increases by 10% and 7%. However, the age of onset (early onset ≤ 65 years and late onset ≥65 years) does not moderate the relationship between high SBP/DBP and AD, showing similar effect sizes for both categories. Related to the measure of BP, this study found that elevated SBP > 160 mmHg was associated with the risk of AD in the young elderly (≤ 65 years), but not in those ≥65 years of age. In this vein, several studies have found that hypertension has different impacts on cognitive function at different ages (19, 22, 110). Current literature indicates that hypertension is a risk factor for cognitive decline in midlife and young old age but may be protective against cognitive decline in late life (22). For example, some authors concluded that high BP at the early age of onset impacted cognitive functions and increased the risk of developing AD in older age (19, 113). Iadecola et al. (114) also found that hypertension in early onset is associated with a higher risk of AD. Therefore, changes in BP may be due to hemodynamic regulation being altered by neurodegenerative processes in the years preceding disease onset (22).

The only variable that moderates the relationship between BP and AD is the region. We observe a higher risk of AD in Asia with SBP >140 mmHg (*RR* = 1.34, 95%CI: 1.096, 1.637, *Z* = 2.854, *p* = 0.004) compared with European (*RR* = 0.99, 95%CI: 0.829, 1.193, *Z* = −0.057, *p* = 0.955) and North America (*RR* = 1.01, 95%CI: 0.866, 1.174, *Z* = 0.109, *p* = 0.913). Therefore, the rate risk of AD in Asia increases by 34%. These results are related to the findings of some studies. During the past four decades, the highest BP measurements worldwide have shifted from high-income countries to low-income countries, such as South Asia and Africa (115), which could explain our results (116, 117). On the one hand, several authors suggest that recent lifestyle changes in Asia countries, such as diet, changing demographics, urbanization, environmental interactions, and other factors, may help explain this relationship (117). On the other hand, one study with data from 90 countries showed that the percentage of people with hypertension receiving treatment increased in both high-income and low- and middle-income countries, but the gap between them widened (118). Moreover, our results also show that the risk of AD related to SBP > 160 mmHg in Europe (*RR* = 0.61, 95%CI: 0.060, 1.159, *Z* = 2.176, *p* = 0.030) and Asia (*RR* = 0.23, 95%CI: 0.067, 0.389, *Z* = 2.771, *p* = 0.006) is significant. However, North America (*RR* = 0.01, 95%CI: −0.318, 0.334, *Z* = 0.047, *p* = 0.962) did not find a significant relationship. Despite these results, the strength of the association between SBP (>160 mmHg) and AD risk is similar in the three regions.

Finally, results do not find differences in the effect size of the association between high SBP and DBP and the risk of AD according to the type of design (cross-sectional and longitudinal). Our results found an association between BP and the risk of AD in both types of studies. However, findings confirm that the relationship between higher SBP and AD is only significant in longitudinal studies and with SBP > 160 mmHg (*RR* = 1.23, 95%CI: 1.067, 1.428, *Z* = 2.834, *p* = 0.005), so the rate risk of AD increases by 23%, while high DBP (>85 and >90 mmHg) is not related to increased AD risk. In this vein, previous work found differences according to the type of design that may result in part from the use of different definitions of hypertension and non-uniform measures of high or low BP. In this study, we use standardized criteria to define BP (SBP > 140/160 mmHg and DBP > 85/90 mmHg) and AD (clinical criteria) which could explain that there are no differences according to the study design. After controlling for this confounding factor, the effect size of longitudinal studies is higher in all the BP and SBP measures, although the differences do not reach significance. Longitudinal studies provide an opportunity to assess the temporal relationship between BP and AD and the length of follow-up remains relevant since hypertension could render individuals more vulnerable to comorbid conditions, such as cerebrovascular disease, that confer greater risk for AD during long periods of follow-up.

However, there are some limitations to our study. The key limitation is that only a small number of studies examined the association between DBP, both types of BP combined, and AD compromising the generalizability of the results. Furthermore, it is likely that due to the procedure used in this meta-meta-analysis, some primary studies were not included. Another challenge was that studies reported outcomes using different metrics (OR, HR, and RR). Likewise, not all the cutoff points established by ISH could be analyzed since the stages of SBP ≥ 130–139 and DBP ≥ 100 could not be defined due to the lack of primary studies. Other confounders may also influence the study's findings. For example, results were not adjusted for other risk variables including cardiovascular disease, stroke, alcohol consumption, smoking, kidney disease, and many others. Also, two studies did not report the mean age of the sample, and they were not included in the moderator analysis. Moreover, the relationship between hypertension and AD could not be thought of as binary but rather as a dynamic one, changing with life stage and disease state. Hence, a single measurement of BP may not accurately reflect the participant's average BP measurements. Additionally, data on the age at the onset of hypertension and years of living with the condition may be important in clarifying temporal relationships between hypertension and AD. Also, we did not examine the potentially modifying impact of antihypertensive therapy on the relationship between hypertension and AD. In addition, another limitation is the absence of studies from South America and Australia. Finally, we did not include educational level as a moderator variable since the external validity of some of the results has been questioned. The primary studies contained in this meta-analysis used very different forms of measurement. For instance, some studies analyzed education using individual (i.e., no formal education, mandatory education, secondary studies, university studies) (79, 88) or community-based samples (i.e., family education level, region, or country) (80, 88), quantitative (linear relation between the number of years of education and the risk of dementia) (81, 83) or qualitative measures (a threshold effect at a given level of education) (86), and composite measures (i.e., socioeconomic status, SES defines education plus income) (67, 119) that show different results. Therefore, we should interpret our results cautiously.

Several strengths of our review of a meta-analysis should be emphasized. First, most prior studies were drawn from general community samples or non-AD-specific studies (vascular dementia, cortical dementia, or dementia in general), whereas the current study relied on AD. Second, we add to the current literature by analyzing 52 primary studies extracted from the previous meta-analysis increasing the statistical power of our results. Third, we analyzed the impact of different moderators (sex, age, study design, region, and measures of SBP/DBP) to explore the influence of other research parameters in the relationship between high SBP and DBP and AD. Finally, we want to focus on effect sizes since the statistical significance should never be interpreted as evidence that an effect had clinical importance. It is important to note that the effect sizes were “relatively small” and the variation is great within the same meta-analysis. Therefore, the clinical significance and practical importance of these results should be considered in relation to the patient's status, goals, and clinician experience.

As a practical implication, this study suggests that high SBP could be a risk factor for AD. There is limited evidence that single cardiovascular risk factors affect AD risk, but the strength of the association is influenced greatly by changing the parameters of the risk factors and in particular by identifying interactions between the factors. Future research should confirm this and determine whether stabilizing BP might be a target to slow or decline the development of AD.

## 5. Conclusion

This study analyzes the association between SBP/DBP/combined BP and the risk of developing AD. A total of five meta-analyses and 52 primary studies were analyzed in this review of meta-analysis. Our study found that SBP is associated with an increased risk of AD by 11%, although no association was found for DBP. Measures of SBP >140, SBP >160, DBP >85, and DBP >90 do not moderate the relationship between SBP and DBP and AD. Moderator analysis (sex, age, study design, region, and measures of SBP/DBP) shows a significant association between high DBP (>90) and AD in women. The age of onset (early-onset AD ≤ 65 years and late-onset AD or senile AD ≥65 years) did not moderate the relationship between SBP and DBP and AD. Finally, regarding the type of study, there were no differences in the association between BP and AD between longitudinal and cross-sectional studies. However, Asian countries showed stronger effect sizes between SBP > 140 and risk of AD than European and North American countries. Future work should use other uncontrolled factors (e.g., cardiovascular diseases, diabetes, and stroke) to explain the relationship between high BP and AD.

## Data availability statement

The original contributions presented in the study are included in the article/Supplementary material, further inquiries can be directed to the corresponding author.

## Author contributions

OS-V and AP-M conceived and designed the analysis, collected the data, contributed data or analysis tools, performed analysis, and wrote the paper. JP-B wrote the paper. SU-L contributed data or analysis tools, performed analysis, and wrote the paper. All authors take full responsibility for the data, the analyses and interpretation, and the conduct of the research, full access to all of the data and the right to publish any and all data. All authors contributed to the article and approved the submitted version.
