# Supplementary material for: Blood pressure and Alzheimer's disease: A review of meta-analysis
Source: Front Neurol. 2023 Jan 11;13:1065335. doi: 10.3389/fneur.2022.1065335 (PMC9874700; doi:10.3389/fneur.2022.1065335)
Supplement: Supplementary file 1 [file Table_1.docx]

SUPPLEMENTAL MATERIAL

| Table complementary 1: Characteristics of primary studies | | | | | | | | | | |
| --- | --- | --- | --- | --- | --- | --- | --- | --- | --- | --- |
|  | **Year^1^** | **Country^2^** | **Age^3^** | **Women^4^ (%)** | **n AD^5^** | **SBP (140-159)^6^** | **SBP (>160)^6^** | **DBP (70-89)** | **DBP >90** | **SBP and DBP^8^** |
| Kivipelto et al. [1] | 2001 | Finland | 50,4 | 61 | 48 | 2,10 (0.84-5.25) |  |  | * | * |
| Morris et al. [2] | 2001 | USA | 63 | 63 | 324/54/78 | 1,03 (0,80-1,32) | 1.13 (0.24-5.35) |  | 1.56 (0.6-4.07) | * |
| McGrath et al.[3] | 2017 | USA | 55 | 53 | 81 | 1,35 (0,84 – 2.17) |  |  | * | * |
| Chiang et al.[4] | 2007 | Taiwan | 57,9 | 41 | 64 | 1,26 (0,64-2,49) |  |  | * | * |
| Kimm et al. [5] | 2011 | Korea | 52,6 | 0 | 282/274 | 1,30 (1,03-1,64) | 1.40 (1.09-1.79) |  | * | * |
|  | 2011 | Korea | 53,6 | 100 | 164/206 | 1,2 (0,38-4,04) | 1.20 (0.96-1.50) |  | * | * |
| Ninomiya et al. [6] | 2011 | Japan | 57,4 | 60 | 6/17 | 0,95 (0,54-1,68) | 0.84 (0.42-1.67) |  | * | * |
| Launer et al.[7] | 2000 | USA | * | 0 | 81/87 | 1,22 (0,37-4,04) |  |  | 1,86 (1,01-3,46) | * |
| Posner et al.[8] | 2002 | USA | * | 55 | 257 | 0,80 (0,42-1,60) |  |  | * | * |
| Verghese et al.[9] | 2003 | USA | * | 32 | 65/65 | 0,68 (0,35-1,32) |  |  | 1,91 (1,05-3,48) | * |
| Tyas et al.[10] | 2001 | Canada | * | * | 35 | 1,14 (0,53-2,45) |  |  | * | * |
| Bermejo et al.[11] | 2010 | Spain | * | 28 | 113 | 2,07 (0,98-4,37) |  |  | * | * |
| Huang et al.[12] | 2014 | China- Taiwan | * | 24 | 612 | 1,3 (1,07-1,59) |  |  | * | * |
| Chu et al.[13] | 2010 | China- Hongkong | 75,1 | 0 | 10 | 1,044 (1,00-1,07) |  |  | * | * |
| Luchsinger et al.[14] | 2005 | USA | 76,2 | 35 | 246 | 1,4 (0,9-2,1) |  |  | * | * |
| Forti et al.[15] | 2010 | Italy | 69,3 | 26 | 18 | 0,77 (0,17-3,52) |  |  | * | * |
| Forti et al.[15] | 2010 | Italy | 79,8 | 34 | 30 | 0,97 (0,39-2,4) |  |  | * | * |
| Song et al.[16] | 2011 | Canada | * | * | 416 | 1.01 (0,75-1,32) |  |  | * | * |
| Raffaitin et al.[17] | 2009 | Franca | * | 30 | 134 | 1,1 (0,6-2,01) |  |  | * | * |
| Muller et al.[18] | 2007 | USA | 76,1 | 34 | 147 | 1,5 (0,9-2,4) |  |  | * | * |
| Lindsay et al.[19] | 2002 | Canada | * | 63 | 194 | 0,88 (0,62-1,27) |  |  | * | * |
| Kivipelto et al.[20] | 2002 | Finland | * | 33 | 48 | 2,6 (1,1-6,6) |  |  | * | * |
| Borenstein et al.[21] | 2005 | Japan | * | * | 90 | 1,79 (0,82-3,89) |  |  | * | * |
| Hayden et al.[22] | 2006 | UK | 77,75 | 66 | 104 | 0,66 (0,43-1,02) |  |  | * | * |
| Kuller et al.[23] | 2003 | USA | * | * | 330 | 0,9 (0,57-1,48) |  |  | * | * |
| Ronnemaa et al.[24] | 2011 | Sweden | 49,6 | * | 127 | 1 (0,99-1,01) |  |  | * | * |
| Annweiler et al.[25] | 2012 | France | 79,84 | * | 70 | 0,7 (0,38-1,29) |  |  | * | * |
| Wang et al.[26] | 2012 | China- Taiwan | * | 26 | 8488 | 1,38 (1,07-1,75) |  |  | * | * |
| Qiu et al. [27] | 2006 | Sweden | * | 37 | 333/87 | 1,32 (0,97-1,81) |  |  | 1,9 (1,36-2,68) | * |
| Olazaran et al.[28] | 2013 | Spain | * | * | 68 | 0,64 (0,39-1,06) |  |  | * | * |
| Becker et al.[29] | 2009 | USA | 77,52 | 31 | 48 | 0,9 (0,48-1,67) |  |  | * | * |
| Dal et al.[30] | 2005 | USA | 66,8 | 0 | 40 | 0.87 (0,46-1,64) |  |  | * | * |
| Dal et al.[30] | 2005 | USA | 64 | 100 | 67 | 0,58 (0,32-1,04) |  |  | * | * |
| Harwood et al.[31] | 1999 | USA | 77,8 | 64 | 202 | 1.50 (1-2,3) |  |  | * | * |
| Harwood et al.[31] | 1999 | USA | 73,75 | 67 | 188 | 0,70 (0,37-1,26) |  |  | * | * |
| Wu et al.[32] | 2003 | China | * | * | 201 | 1,97 (1,09-3,5) |  |  | * | * |
| Brayne et al.[33] | 1998 | UK | * | 68 | 18 | 0,78 (0,38-1,65) |  |  | * | * |
| Mendez et al.[34] | 1992 | USA | * | 55 | 50 | 0,94 (0,02-54,26) |  |  | * | * |
| French et al.[35] | 1985 | USA | * | 0 | 76 | 0,93 (0,41-2,09) |  |  | * | * |
| Kokmen et al.[36] | 1991 | USA | * | * | 203 | 0,7 (0,38-1,3) |  |  | * | * |
| Foroughan et al.[37] | 2008 | Iran | 70 | 43 | 42 | 1,712 (1,08-2,7) |  |  | * | * |
| Roberts et al.[38] | 2006 | USA | * | 100 | 151 | 1,07 (0,71-1,63) |  |  | * | * |
| Kondo et al.[39] | 1994 | Japan | * | * | 60 | 1,5 (1,10-2,1) |  |  | * | * |
| Suhanov et al.[40] | 2006 | Russia | 69,25 | 72 | 127 | 1,8 (1-10,3) |  |  | * | * |
| Graves et al.[41]^9^ | 1990 | USA | * | 68 | 18 | 0,5 (0,23-1,07) |  |  | * | * |
| Tsolaki et al.[42]^9^ | 1997 | Greek | * | 54 | 65 | 0,46 (0,31-0,67) |  |  | * | * |
| Imfeld et al.[43]^9^ | 2012 | UK | * | 69 | 3541 | 0,68 (0,63-0,73) |  |  | * | * |
| Joas et al.[44] | 2012 | Sweden | 45 | 100 | 79 | 4,92 (1,35-8,48) |  |  | * | * |
| Qiu et al. a[45] | 2010 | Suecia | 81.5±5 | 75 | 150 |  | 1.84 (1.06-3.18) |  |  |  |
|  | 2010 | Suecia | * | * | 124/41 | 1.47 (1.02-2.12) |  |  | 0.78 (0.54-1.12) | * |
| Li et al. [46] | 2007 | USA | 65-74 | 59 | 4//37/22/4 | 0.48 (0.15-1.57) | 0.94 (0.62-1.42) | 1.71 (0.98-2.97) | 0.82 (0.29-2.35) | * |
|  | * | * | * | * | 19/14 | 1.47 (0.80-2.71) | 1.38 (0.71-2.70) |  | * | * |
| Li et al. [46] | 2007 | USA | 75-84 | 59 | 31/28/7 | 0.60 (0.38-0.92) |  | 0.96 (0.63-1.47) | 0.73 (0.34-1.59) | * |
|  | 2007 | USA | 75-84 | 59 | 37 |  | 0.94 (0.62-1.42) |  |  |  |
| Ruitenberg et al.[47] | 2001 | USA | 69,7 | 30 | 107 | * |  |  | 0,9 (0,72-1,13) | * |
| Shah et al.[48] | 2006 | Sweden | 75 | * | 151 | * |  |  | 1 (0,99-1,01) | * |
| Qiu et al.[49] | 2003 | Sweden | >75 | * | 75 | * |  |  | * | 1,24 (0,85-1,83) |
| Stewart et al.[50] | 2009 | Japanese | 40-44 | * | 35 | * |  |  | * | 0,89 (0,57-1,40) |
| Treiber et al.[51] | 2008 | USA | >60 | * | 65 | * |  |  | * | 1,18 (0,90-1,54) |
| Hassing et al.[52] | 2009 | Sweden | >45-65 | * | 36 | * |  |  | * | 0,84 (0,64-1,09) |

Note:

^5^ Sample: AD: AD cases SBP/ AD cases DBP

^6^ SBP: Systolic Blood Pressure. *CI*: 95% confidence interval; *RR*: Risk Ratio

^7^ DBP: Diastolic Blood pressure. *CI*: 95% confidence interval; *RR*: Risk Ratio

^8^ SBP: Systolic Blood Pressure; DBP: Diastolic Blood pressure. *CI*: 95% confidence interval; *RR*: Risk Ratio

^9^ Outliers studies.

**REFERENCES**

[1] Kivipelto M, Helkala E-L, Laakso MP, Hänninen T, Hallikainen M, Alhainen K, Soininen H, Tuomilehto J, Nissinen A (2001) Midlife vascular risk factors and Alzheimer’s disease in later life: longitudinal, population based study. *Bmj* **322**, 1447–1451. https://doi.org/10.1136/bmj.322.7300.1447

[2] Morris MC, Scherr PA, Hebert LE, Glynn RJ, Bennett DA, Evans DA (2001) Association of incident Alzheimer disease and blood pressure measured from 13 years before to 2 years after diagnosis in a large community study. *Arch Neurol* **58**, 1640–1646. https://doi.org/10.1001/archneur.58.10.1640

[3] McGrath ER, Beiser AS, DeCarli C, Plourde KL, Vasan RS, Greenberg SM, Seshadri S (2017) Blood pressure from mid‐to late life and risk of incident dementia. *Neurology* **89**, 2447–2454. https://doi.org/10.1212/WNL.0000000000004741

[4] Chiang C-J, Yip P-K, Wu S-C, Lu C-S, Liou C-W, Liu H-C, Liu C-K, Chu C-H, Hwang C-S, Sung S-F (2007) Midlife risk factors for subtypes of dementia: a nested case-control study in Taiwan. *Am J Geriatr Psychiatry* **15**, 762–771. https://doi.org/10.1097/JGP.0b013e318050c98f

[5] Kimm H, Lee P, Shin Y, Park K, Jo J, Lee Y, Kang H, Jee S (2011) Mid-life and late-life vascular risk factors and dementia in Korean men and women. *Arch Gerontol Geriatr* **52**, e117–e122. https://doi.org/10.1016/j.archger.2010.09.004

[6] Ninomiya T, Ohara T, Hirakawa Y, Yoshida D, Doi Y, Hata J, Kanba S, Iwaki T, Kiyohara Y (2011) Midlife and late-life blood pressure and dementia in Japanese elderly: the Hisayama study. *Hypertension* **58**, 22–28. https://doi.org/10.1161/HYPERTENSIONAHA.110.163055

[7] Launer LJ, Ross GW, Petrovitch H, Masaki K, Foley D, White LR, Havlik RJ (2000) Midlife blood pressure and dementia: the Honolulu–Asia aging study☆. *Neurobiol Aging* **21**, 49–55. https://doi.org/10.1016/S0197-4580(00)00096-8

[8] Posner HB, Tang M-X, Luchsinger J, Lantigua R, Stern Y, Mayeux R (2002) The relationship of hypertension in the elderly to AD, vascular dementia, and cognitive function. *Neurology* **58**, 1175–1181. https://doi.org/10.1212/WNL.58.8.1175

[9] Verghese J, Lipton R, Hall C, Kuslansky G, Katz M (2003) Low blood pressure and the risk of dementia in very old individuals. *Neurology* **61**, 1667–1672. https://doi.org/10.1212/01.WNL.0000098934.18300.BE

[10] Tyas SL, Manfreda J, Strain LA, Montgomery PR (2001) Risk factors for Alzheimer’s disease: a population-based, longitudinal study in Manitoba, Canada. *Int J Epidemiol* **30**, 590–597. https://doi.org/10.1093/ije/30.3.590

[11] Bermejo-Pareja F, Benito-León J, Louis ED, Trincado R, Carro E, Villarejo A, De La Cámara AG (2010) Risk of incident dementia in drug-untreated arterial hypertension: a population-based study. *J Alzheimers Dis* **22**, 949–958. https://doi.org/10.3233/JAD-2010-101110

[12] Huang C-C, Chung C-M, Leu H-B, Lin L-Y, Chiu C-C, Hsu C-Y, Chiang C-H, Huang P-H, Chen T-J, Lin S-J (2014) Diabetes mellitus and the risk of Alzheimer’s disease: a nationwide population-based study. *PloS One* **9**, e87095. https://doi.org/10.1371/journal.pone.0087095

[13] Chu L-W, Tam S, Wong RL, Yik P-Y, Song Y, Cheung BM, Morley JE, Lam KS (2010) Bioavailable testosterone predicts a lower risk of Alzheimer’s disease in older men. *J Alzheimers Dis* **21**, 1335–1345. https://doi.org/10.3233/JAD-2010-100027

[14] Luchsinger J, Reitz C, Honig LS, Tang M-X, Shea S, Mayeux R (2005) Aggregation of vascular risk factors and risk of incident Alzheimer disease. *Neurology* **65**, 545–551. https://doi.org/10.1212/01.wnl.0000172914.08967.dc

[15] Forti P, Pisacane N, Rietti E, Lucicesare A, Olivelli V, Mariani E, Mecocci P, Ravaglia G (2010) Metabolic syndrome and risk of dementia in older adults. *J Am Geriatr Soc* **58**, 487–492. https://doi.org/10.1111/j.1532-5415.2010.02731.x

[16] Song X, Mitnitski A, Rockwood K (2011) Nontraditional risk factors combine to predict Alzheimer disease and dementia. *Neurology* **77**, 227–234. https://doi.org/10.1212/WNL.0b013e318225c6bc

[17] Raffaitin C, Gin H, Empana J-P, Helmer C, Berr C, Tzourio C, Portet F, Dartigues J-F, Alpérovitch A, Barberger-Gateau P (2009) Metabolic syndrome and risk for incident Alzheimer’s disease or vascular dementia: the Three-City Study. *Diabetes Care* **32**, 169–174. https://doi.org/10.2337/dc08-0272

[18] Muller M, Tang M-X, Schupf N, Manly JJ, Mayeux R, Luchsinger JA (2007) Metabolic syndrome and dementia risk in a multiethnic elderly cohort. *Dement Geriatr Cogn Disord* **24**, 185–192. https://doi.org/10.1159/000105927

[19] Lindsay J, Laurin D, Verreault R, Hébert R, Helliwell B, Hill GB, McDowell I (2002) Risk factors for Alzheimer’s disease: a prospective analysis from the Canadian Study of Health and Aging. *Am J Epidemiol* **156**, 445–453. https://doi.org/10.1093/aje/kwf074

[20] Kivipelto M, Helkala E-L, Laakso MP, Hänninen T, Hallikainen M, Alhainen K, Iivonen S, Mannermaa A, Tuomilehto J, Nissinen A (2002) Apolipoprotein E ε4 allele, elevated midlife total cholesterol level, and high midlife systolic blood pressure are independent risk factors for late-life Alzheimer disease. *Ann Intern Med* **137**, 149–155. https://doi.org/10.7326/0003-4819-137-3-200208060-00006

[21] Borenstein AR, Wu Y, Mortimer JA, Schellenberg GD, McCormick WC, Bowen JD, McCurry S, Larson EB (2005) Developmental and vascular risk factors for Alzheimer’s disease. *Neurobiol Aging* **26**, 325–334. https://doi.org/10.1016/j.neurobiolaging.2004.04.010

[22] Hayden KM, Zandi PP, Lyketsos CG, Khachaturian AS, Bastian LA, Charoonruk G, Tschanz JT, Norton MC, Pieper CF, Munger RG (2006) Vascular risk factors for incident Alzheimer disease and vascular dementia: the Cache County study. *Alzheimer Dis Assoc Disord* **20**, 93–100. https://doi.org/10.1097/01.wad.0000213814.43047.86

[23] Kuller LH, Lopez OL, Newman A, Beauchamp NJ, Burke G, Dulberg C, Fitzpatrick A, Fried L, Haan MN (2003) Risk factors for dementia in the cardiovascular health cognition study. *Neuroepidemiology* **22**, 13–22. https://doi.org/10.1159/000067109

[24] Rönnemaa E, Zethelius B, Lannfelt L, Kilander L (2011) Vascular risk factors and dementia: 40-year follow-up of a population-based cohort. *Dement Geriatr Cogn Disord* **31**, 460–466. https://doi.org/10.1159/000330020

[25] Annweiler C, Rolland Y, Schott AM, Blain H, Vellas B, Herrmann FR, Beauchet O (2012) Higher vitamin D dietary intake is associated with lower risk of Alzheimer’s disease: a 7-year follow-up. *J Gerontol Ser Biomed Sci Med Sci* **67**, 1205–1211. https://doi.org/10.1093/gerona/gls107

[26] Wang K-C, Woung L-C, Tsai M-T, Liu C-C, Su Y-H, Li C-Y (2012) Risk of Alzheimer’s disease in relation to diabetes: a population-based cohort study. *Neuroepidemiology* **38**, 237–244. https://doi.org/10.1159/000337428

[27] Qiu C, Winblad B, Marengoni A, Klarin I, Fastbom J, Fratiglioni L (2006) Heart failure and risk of dementia and Alzheimer disease: a population-based cohort study. *Arch Intern Med* **166**, 1003–1008. https://doi.org/10.1001/archinte.166.9.1003

[28] Olazaran J, Trincado R, Bermejo-Pareja F (2013) Cumulative effect of depression on dementia risk (2013). *Int J Alzheimer’s Dis* **2013**. https://doi.org/10.1155/2013/457175

[29] Becker JT, Chang Y-F, Lopez OL, Dew MA, Sweet RA, Barnes D, Yaffe K, Young J, Kuller L, Reynolds CF (2009) Depressed Mood is Not a Risk Factor for Incident Dementia in a Community-Based Cohort. *Am J Geriatr Psychiatry* **17**, 653–663. https://doi.org/10.1097/JGP.0b013e3181aad1fe

[30] Dal Forno G, Palermo MT, Donohue JE, Karagiozis H, Zonderman AB, Kawas CH (2005) Depressive symptoms, sex, and risk for Alzheimer’s disease. *Ann Neurol* **57**, 381–387. https://doi.org/10.1002/ana.20405

[31] Harwood DG, Barker WW, Loewenstein DA, Ownby RL, George-Hyslop PS, Mullan M, Duara R (1999) A cross-ethnic analysis of risk factors for AD in white Hispanics and white non-Hispanics. *Neurology* **52**, 551–551. https://doi.org/10.1212/WNL.52.3.551

[32] Wu C, Zhou D, Wen C, Zhang L, Como P, Qiao Y (2003) Relationship between blood pressure and Alzheimer’s disease in Linxian County, China. *Life Sci* **72**, 1125–1133. https://doi.org/10.1016/S0024-3205(02)02367-6

[33] Brayne C, Gill C, Huppert FA, Barkley C, Gehlhaar E, Girling DM, O’Connor DW, Paykel ES (1998) Vascular risks and incident dementia: results from a cohort study of the very old. *Dement Geriatr Cogn Disord* **9**, 175–180. https://doi.org/10.1159/000017043

[34] Mendez MF, Underwood KL, Zander BA, Mastri AR, Sung JH, Frey WH (1992) Risk factors in Alzheimer’s disease: a clinicopathologic study. *Neurology* **42**, 770–770. https://doi.org/10.1212/WNL.42.4.770

[35] French LR, Schuman LM, Mortimer JA, Hutton JT, Boatman RA, Christians B (1985) A case-control study of dementia of the Alzheimer type. *Am J Epidemiol* **121**, 414–421. https://doi.org/10.1093/oxfordjournals.aje.a114013

[36] Kokmen E, Beard CM, Chandra V, Offord KP, Schoenberg BS, Ballard DJ (1991) Clinical risk factors for Alzheimer’s disease: a population-based case-control study. *Neurology* **41**, 1393–1393. https://doi.org/10.1212/WNL.41.9.1393

[37] Foroughan M, Farahani ZG, Shariatpanahi M, Vaezinejad M, Akbari Kamerani AA, Sheikhvatan M (2008) Risk factors of Alzheimer’s disease among Iranian population. *Curr Alzheimer Res* **5**, 70–72. https://doi.org/10.2174/156720508783884594

[38] Roberts RO, Cha RH, Knopman DS, Petersen RC, Rocca WA (2006) Postmenopausal estrogen therapy and Alzheimer disease: overall negative findings. *Alzheimer Dis Assoc Disord* **20**, 141–146. https://doi.org/10.1097/00002093-200607000-00004

[39] Kondo K, Niino M, Shido K (1994) A case-control study of Alzheimer’s disease in Japan–significance of life-styles. *Dement Geriatr Cogn Disord* **5**, 314–326. https://doi.org/10.1159/000106741

[40] Suhanov AV, Pilipenko PI, Korczyn AD, Hofman A, Voevoda MI, Shishkin SV, Simonova GI, Nikitin YP, Feigin VL (2006) Risk factors for Alzheimer’s disease in Russia: a case–control study. *Eur J Neurol* **13**, 990–995. https://doi.org/10.1111/j.1468-1331.2006.01391.x

[41] Graves AB, White E, Koepsell TD, Reifler BV, Van Belle G, Larson EB, Raskind M (1990) A case‐control study of Alzheimer’s disease. *Ann Neurol Off J Am Neurol Assoc Child Neurol Soc* **28**, 766–774. https://doi.org/10.1002/ana.410280607

[42] Tsolaki M, Fountoulakis K, Chantzi E, Kazis A (1997) Risk factors for clinically diagnosed Alzheimer’s disease: a case-control study of a Greek population. *Int Psychogeriatr* **9**, 327–341. https://doi.org/10.1017/S104161029700447X

[43] Imfeld P, Bodmer M, Jick SS, Meier CR (2012) Metformin, other antidiabetic drugs, and risk of Alzheimer’s disease: a population‐based case–control study. *J Am Geriatr Soc* **60**, 916–921. https://doi.org/10.1111/j.1532-5415.2012.03916.x

[44] Joas E, Bäckman K, Gustafson D, Östling S, Waern M, Guo X, Skoog I (2012) Blood pressure trajectories from midlife to late life in relation to dementia in women followed for 37 years. *Hypertension* **59**, 796–801. https://doi.org/10.1161/HYPERTENSIONAHA.111.182204

[45] Qiu C, Xu W, Winblad B, Fratiglioni L (2010) Vascular risk profiles for dementia and Alzheimer’s disease in very old people: a population-based longitudinal study. *J Alzheimers Dis* **20**, 293–300. https://doi.org/10.3233/JAD-2010-1361

[46] Li G, Rhew IC, Shofer JB, Kukull WA, Breitner JC, Peskind E, Bowen JD, McCormick W, Teri L, Crane PK (2007) Age‐varying association between blood pressure and risk of dementia in those aged 65 and older: a community‐based prospective cohort study. *J Am Geriatr Soc* **55**, 1161–1167. https://doi.org/10.1111/j.1532-5415.2007.01233.x

[47] Ruitenberg A, Skoog I, Ott A, Aevarsson O, Witteman JC, Lernfelt B, van Harskamp F, Hofman A, Breteler MM (2001) Blood pressure and risk of dementia: results from the Rotterdam study and the Gothenburg H-70 Study. *Dement Geriatr Cogn Disord* **12**, 33–39. https://doi.org/10.1159/000051233

[48] Shah RC, Wilson RS, Bienias JL, Arvanitakis Z, Evans DA, Bennett DA (2006) Relation of blood pressure to risk of incident Alzheimer’s disease and change in global cognitive function in older persons. *Neuroepidemiology* **26**, 30–36. https://doi.org/10.1159/000089235

[49] Qiu C, Winblad B, Viitanen M, Fratiglioni L (2003) Pulse pressure and risk of Alzheimer disease in persons aged 75 years and older: a community-based, longitudinal study. *Stroke* **34**, 594–599. https://doi.org/10.1161/01.STR.0000060127.96986.F4

[50] Stewart R, Xue Q-L, Masaki K, Petrovitch H, Ross GW, White LR, Launer LJ (2009) Change in blood pressure and incident dementia: a 32-year prospective study. *Hypertension* **54**, 233–240. https://doi.org/10.1161/HYPERTENSIONAHA.109.128744

[51] Treiber KA, Lyketsos CG, Corcoran C, Steinberg M, Norton M, Green RC, Rabins P, Stein DM, Welsh-Bohmer KA, Breitner JC (2008) Vascular factors and risk for neuropsychiatric symptoms in Alzheimer’s disease: the Cache County Study. *Int Psychogeriatr* **20**, 538–553. https://doi.org/10.1017/S1041610208006704

[52] Hassing LB, Dahl AK, Thorvaldsson V, Berg S, Gatz M, Pedersen NL, Johansson B (2009) Overweight in midlife and risk of dementia: a 40-year follow-up study. *Int J Obes* **33**, 893–898. https://doi.org/10.1038/ijo.2009.104
